# Prevalence and predictors of postpartum depression in Riyadh, Saudi Arabia: A cross sectional study

**DOI:** 10.1371/journal.pone.0228666

**Published:** 2020-02-10

**Authors:** Raneem Seif Al Nasr, Kareemah Altharwi, Maria Seraj Derbah, Salma Omar Gharibo, Samah Abdulsallam Fallatah, Sara Ghallab Alotaibi, Khattam Alhumidi Almutairi, Syed Mohammed Basheeruddin Asdaq

**Affiliations:** College of Pharmacy, Al Maarefa University, Riyadh, Saudi Arabia; Universita Cattolica del Sacro Cuore Sede di Roma, ITALY

## Abstract

**Objective:**

Postpartum depression (PPD) is inversely correlated with women’s functioning, marital and personal relationships, mother-infant interaction quality, and children’s social, behavioural, and cognitive development. The purpose of this study was to determine the prevalence of postpartum depression (PPD) in Riyadh and correlate them with possible predictors by a cross-sectional approach.

**Methods:**

In this study, 174 mothers receiving treatments in different hospitals of Riyadh completed self-administered measures of the Edinburgh Postnatal Depression Scale (EPDS) along with a list of probable predictors. The data was analyzed by logistic regression analysis using SPSS-IBM 25.

**Results:**

Of 174 participants of the study, 38.50% (n = 67) reported postpartum depression. Around (115) of the participants were in an age group of 25–45 years with most of them highly educated (101) but unemployed (136). Significant association was noted between occurrence of PPD with unsupportive spouse (*P* value = 0.023) and recent stressful life events (*P* value = 0.003). The significant predictors for PPD were unsupportive spouse (OR = 4.53, *P* = 0.049), recent stressful life events (OR = 2.677, *P =* 0.005), and Caesarean section as a mode of delivery (OR = 1.958, *P* = 0.049)

**Conclusion:**

The prevalence of PPD among the study participants was high, especially those with recent stressful life event and unsupportive spouse. To promote health and wellbeing of mothers, it was recommended to screen all high-risk mothers for PPD, when they visit hospitals for postnatal follow ups. Prevention of PPD is not only essential for wellbeing of mothers but it is important to provide good conducive atmosphere for the new born.

## 1. Introduction

Depression is a common disorder worldwide, with more than 300 million people affected [1]. According to American Psychiatric Association (APA), major depressive disorder (MDD) is manifested with a period of at least two weeks of low mood, or loss of interest [2]. According to the world health organization (WHO), depression was ranked fourth at the start of this century and is expected to be the second by 2020 [3].

One of most common type of depression in women across the world is postpartum depression [4]. Postpartum depression (PPD) is a non-psychotic depressive disorder that is classified by the diagnostic and statistical manual of mental disorders as an episode of major depression with onset within 4 weeks of child birth [2]. The international prevalence of maternal PPD was estimated to be 13%, however, the rate of its prevalence was estimated to be based on screening tools/methods used, screening period and country in which the study was conducted [5]. In the absence of treatment, PPD may cause suicide and rarely infanticide [6]. PPD is different from baby blue syndrome, which usually resolves in a few days to one week [7].

Postpartum depression is inversely associated with women’s functioning, marital and personal relationships, mother-infant interaction quality, and children’s social, behavioral, and cognitive development [8]. Maternal depression is referred to as the “thief that steals motherhood” [9] because it reduces mothers’ enjoyment in her maternal role. Depressed mothers often show minimum responsiveness in interactions with their infants [10] and fail to meet their social-emotional needs [11]. Research suggests that infants of mothers with PPD compared to those of non-depressed mothers are more likely to be abused and neglected [12], fail to thrive [13], be hospitalized with health problems such as asthma [14], and have sleeping disturbances [15]. Also, depressed mothers are more likely to have low intensity breastfeeding, add cereal to formula and start solid food earlier [16]. As a result, their infants will receive less immune protection and other benefits of breastfeeding. PPD adversely affects infants’ cognitive development and learning [17]. Long-term impacts of PPD on children’s development have been demonstrated by lower vocabulary and cognition scores [18].

The Edinburgh postnatal depression scale (EPDS) is one of the most common tool for assessing depression in prevalent studies reported till date on PPD. There is high variation among reported research on the prevalence of PPD. A study in Italy showed only 4.7% [19], and while a study of Brazil reported 10.8% [20] of prevalence of PPD. Studies done in Turkey, Malaysia and China reported only 9.1% [21], 14.3% [22] and 27.37% [23], respectively, of PPD prevalence.

Large cross-sectional studies were conducted in Middle Eastern countries. The prevalence rate was not consistent in all studies even though they share similar socioeconomic conditions, such as only 10% PPD prevalence was reported in a study done in Sharjah, UAE [24], whereas, a study in Qatar found 17.6% of PPD prevalence [25]. Further, a report from Lebanon explained only 12.8% of PPD [26], whereas, an epidemiological study conducted among rural women in Minia, Egypt showed 49.5% of PPD occurrence [27]. A study published in 2014 from Saudi Arabia demonstrated 17.8% of PPD prevalence in Dammam region [28]. While in the same year, another study in Riyadh showed a prevalence of PPD of 33.2% [29] A more recent study in Riyadh reiterated high prevalence of PPD in Riyadh [30].

The variation in prevalence among different population might be due to the disparities in health care settings or social and geographical differences. The meta-analyses carried out during mid-nineties triggered a debate on the magnitude of relationship between postpartum depression and significant risk factors [31, 32]. The exact reason for the induction of PPD remain unknown; however, lack of social support [33], marital conflict [34], prior history of depression [35], lack of breastfeeding [36] and recent stressful life events [37] were some of the risk factors reported by researchers. Additionally, unemployment, low level of education and undesired pregnancy [38–40] were also reported as factors associated with increased risk of developing PPD. There is a need for validation of risk factors for PPD in communities with distinctive cultural values, such as Saudi Arabia. There is a strong influence of religion in Saudi Arabian community. Therefore, values of their religion on social integrity and mutual care, with good knowledge on triggers of PPD may help community to take appropriate preventive care of new mothers.

The existing literature, although limited, suggest that PPD is common across female Arabian population. However, low sample size, collection of data from single site and dearth of studies that compare PPD prevalence with its risk factors in Saudi Arabian context warrant the impetus for further research. Therefore, this study was aimed to determine the prevalence of PPD and explore its risk factors in female study participants of Riyadh region of Saudi Arabia.

## 2. Materials and methods

### 2.1 Study design

This is cross sectional study carried out during January to April 2018 using Arabic version of validated pretested questionnaire of Edinburgh Postnatal Depression Scale (EPDS).

### 2.2 Location and population

To get the best representation of the residents of Riyadh, various types of health care settings were selected in this study. Health care services in Riyadh are offered by both government hospitals and private health centres. The government hospitals usually provide health care services to the citizens of Saudi Arabia and employees of ministries, while, private hospitals offer services to all residents. Around 25% of the study participants were recruited from King Saud Medical City (a government tertiary care center of Riyadh with large daily patient flow to Gynecology and Obstetrics unit), whereas, rest of the participants were either from government polyclinics or private health centers representing diverse population of Riyadh. The polyclinic chosen as sampling sites in our study were Al- warood medical center, Bader primary care, Aldar Albaidah primary health care center, Pioneers Specialist Medical Center, and Al azhar hospital.

All sampling sites chosen in this study have active and fully functional postpartum care unit. Using the convenience sampling approach, women who visited for a routine postpartum follow-up visit and immunization of their newborns were surveyed. Those who were undergoing treatment for psychological problems or mothers whose babies were diagnosed with serious health problems, stillborn, or experienced intrauterine fetal death were excluded. With a 13% prevalence of PPD reported earlier by meta-analysis paper [32], as well as based on the results of large number of other studies, considering 15,86,524 as the female population of Riyadh in an age group of 16–45 years (https://www.stats.gov.sa/en/5680), 5% as precision percentage and a 95% confidence level, the required sample size was calculated to be 174 by http://sampsize.sourceforge.net. This study included 209 mothers from different health centers, by proportionate allocation to the population served by each health center. However, due to missing data of some of the important elements of questionnaire, 35 samples were removed and therefore the study samples used for our analysis were only 174.

### 2.3 Data collection

The data collection team was composed of female Pharm.D students from the College of Pharmacy, Al-Maarefa University, Riyadh. This team was trained by their supervisor on method of introduction of subject to the participants in the targeted setting, present them with the survey forms, and collect their responses. A pilot study was conducted in King Saud Medical City, Riyadh. The participants were interviewed using Arabic version of the Edinburgh Postnatal Depression Scale (EPDS) [37]. If the study participant was illiterate, the data collector conducted the survey through a face-to-face interview. The EPDS scale was validated and translated into 18 languages, including Arabic by department of health, Government of Western Australia (https://www.mcpapformoms.org/Docs/Edinburgh Depression Scale Translated Government of Western Australia Department of Health.pdf). Each survey filled both sides of a single sheet of paper and consisted of a demographics section and the standard Edinburgh Postnatal Depression Scale (EPDS). The projected time of administration and completion of the survey was approximately 10 min.

Demographic Information: the survey inquired about the age, weeks post-parturition, level of education, employment status, income level, supportive/non supportive spouse, desired/undesired pregnancy, mode of delivery, number of previous deliveries, history of depression, postpartum phase (upto 4 weeks-phase 1; >4–7 weeks-Phase 2 and 8–24 weeks-Phase 3). breast feeding, use of contraceptives post-parturition and the recent stressful life events.

Edinburgh Postnatal Depression Scale (EPDS): It is composed of 10 items to assess the mother’s emotional experience over the past 7 days. Responses were scored 0–3 indicating the severity of manifestations, with a maximum score of 30. The cumulative depression score for each participants was calculated. Several validation studies recommended different cut-off score for optimal sensitivity. Initially, the cutoff point was established at eleven in Guedeney and Fermanian’s study [41]. A study done in Iran by Montazeri et al, [42] proposed three interpretive categories of scoring EPDS scale: those with a score of ≥13 were to be considered postnatally depressed, while scores of 10–12 represented “borderline” and 0–9 “not depressed”. Cox and Holden [43] suggest that these differences are due to variation in population size, timing of administration of the EPDS, differences in expression and cultural variations. Further, some reports available from Saudi Arabia used EPDS scale with ≥12 cut-off for depression [37, 43]. A more recent report by Almutairi [30] used 13 or more cut-off for depression. Therefore, in the present study, mothers who scored ≥13 on the EPDS were considered to be possible PPD sufferers and those who scored <13 were considered normal.

### 2.4 Ethical considerations

Ethical approval to conduct this study was obtained from the Institutional Review Board Committee of King Saud Medical City, Riyadh. All necessary approvals were taken from other study location in addition to approval from the Research Committee of College of Pharmacy, Al-Maarefa University, Riyadh. Participation was completely voluntary, and anonymous. The survey was conducted only after the purpose, procedure, benefits, and potential risks were explained to the respondents, and they consented by checking the “agree to participate” box on the covering page of survey form.

### 2.5 Statistics

The data collected was entered into SPSS IBM statistical package (version 25). Univariate descriptive analysis of the socio-demographic characteristics of the study sample and bivariate analysis, using the Pearson Chi-square test were conducted. The predictors of PPD were determined using binary logistic regression analysis (S1 File). A *P*-value of less than 0.05 was considered to be significant.

## 3. Results

### 3.1 Sample characteristics

During the course of this study in different hospitals of Riyadh, based on the inclusion criteria mentioned above 279 mothers were approached, of them 209 agreed to participate (response rate; 75%). However, the information given by 35 participants were not complete and hence data from only 174 participants was considered for analysis. Of the 174 participants, 34% (n = 59) were in an age group of 16–25 years, while the rest (66%, n = 115) were more than 25 upto 45 years of age. Most of the women of this study were highly educated (58% graduates or above, 58%, n = 101) with low employment rate (78% not working, n = 136) and hence lower income range (63%, n = 109). All the participants of this study were married and living with their spouse. Around 6.5% (n = 11) were not comfortable with the unsupportive attitude of their spouse. Majority of the surveyors (77%, n = 134) had a desired pregnancy, while, approximately 57% of the surveyed women had a spontaneous vaginal delivery and about 43% underwent a cesarean-section (C-section) delivery. Nearly 77% (n = 134) of the mothers were breastfeeding their newborns and 60% (n = 104) of the participants acknowledged the use of some method of contraception. Around 86% (n = 149) of them had three or less than three deliveries, while the rest had more than three deliveries (14%, n = 25). Only 25 (14%) surveyors admitted past history of depression. Around 39% (67) of them had a recent stressful event in their life such as loss of job (15), increased financial obligation (12), fight with someone at home (9), death of the loved one (4), with 24 of them did not mentioned any specific reason and three stated 'don't like to write'. Only 46 (26%) mothers of this study were in phase 1 (upto four weeks postpartum), whereas, rest of them (74%, n = 128) were in other phases (>4 weeks upto 24 weeks).

### 3.2 Prevalence of postpartum depression

**Fig 1:**
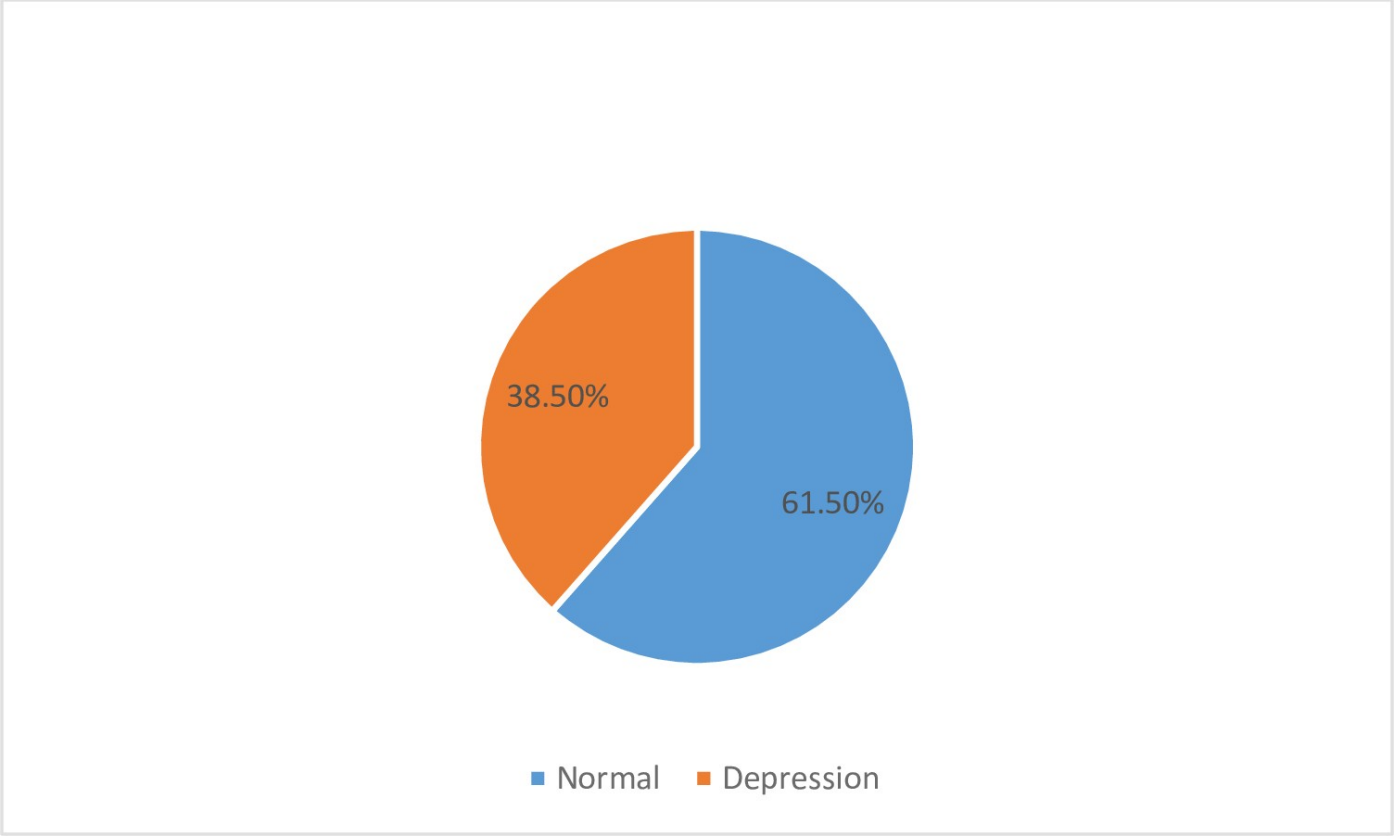
Prevalence of Postpartum depression in Riyadh.

**Fig 2:**
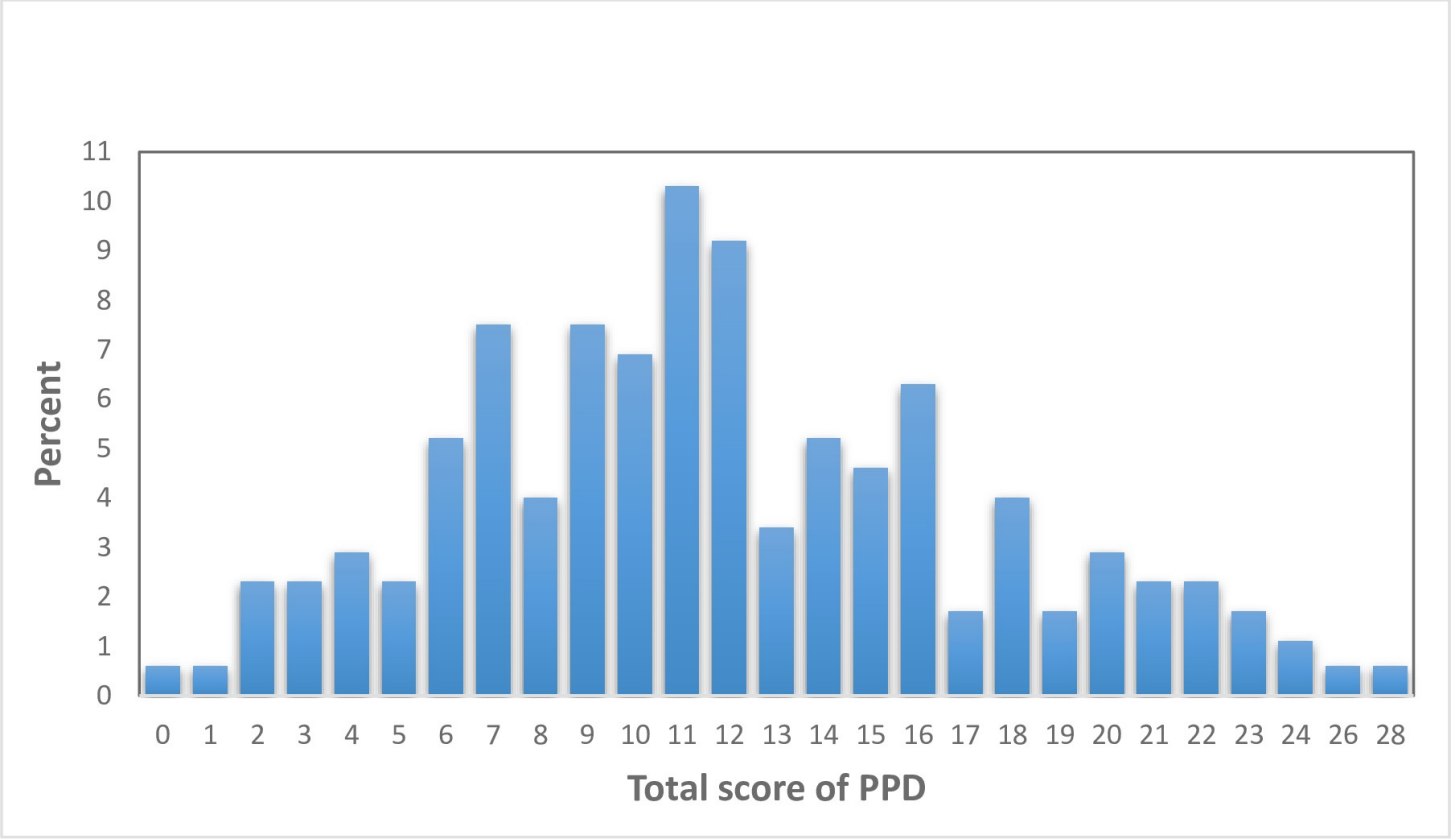
Distribution of study participants with PPD score.

Of those surveyed, 38.50% (n = 67) reported postpartum depression (≥ 13 score in EPDS scale), whereas, 61.50% (n = 175) of them were found normal with a score of less than 13 in EPDS scale (Fig 1). Fig 2 illustrates the distribution of PPD scores.

### 3.3 Association of PPD with socio-demographic characters

As shown in Table 1, there was no significant difference between the occurrence of PPD and socio-demographic characteristics. Only 35.6% of the mothers in an age group of 16–25 years were found with PPD compared to 40% in higher age group (> 25 years) Further, 37% and 40% of the participants from low education and high education cohorts, respectively, had PPD. Furthermore, out of all surveyors with employment, 40% were confirmed with PPD, whereas in unemployed batch, only 38% were diagnosed with PPD indicating no significant impact of employment on development of depression. However, compared to a cohort with higher income (> 8000 SAR/month), presence of depression in mothers with lower income (≤ 8000 SAR/month) was high (40.4% Vs 35.4%).

**Table 1:** Association between postpartum depression and the socio-demographic characteristics.

Socio-demographic characters	Normal*	Depression*	Total*	*P* value**
**1) Age**				
a) 16–25 years	38 (64.4%)	21 (35.6%)	59 (100%)	0.572
b) > 25 years	69 (60%)	46 (40%)	115 (100%)	
**2) Level of education**				
a) Low education^**#**^	46 (63%)	27 (37%)	73 (100%)	0.726
b) high education^**##**^	61(60.4%)	40 (39.6%)	101 (100%)	
**3) Employment status**				
a) Yes	23 (60.5%)	15 (39.5%)	38 (100%)	0.890
b) No	84 (61.8%)	52 (38.2%)	136 (100%)	
**4) Income level**				
a) ≤ 8000 SAR	65 (59.6%)	44 (40.4%)	109(100%)	0.514
b) > 8000 SAR	42(64.6%)	23 (35.4%)	65 (100%)	

*Values are given in frequency (percentage)

**P value is calculated by Pearson Chi-square test

^#^low education: Secondary, intermediate and elementary education

^**##**^High education: Graduate and above

### 3.4 Association of PPD with Psycho-social characters

Significant association was noted (Table 2) between occurrence of PPD and unsupportive spouse (*P* value = 0.023). Further, recent stressful life events contributed significantly (*P* value = 0.003) for the development of PPD in sample population. Even though, no significant association was noted between presence of PPD and history of depression, there was higher degree of depressive cases in patients who had prior ‘history of depression’ compared to the patients with ‘no depression history’ (44% Vs 37.6%).

**Table 2:** Association between postpartum depression and Psycho-social characters.

Psycho-social characters	Normal*	Depression*	Total*	*P* value**
**Spouse:**				0.023^#^
a) Supportive	104 (63.8%)	59 (36.2%)	163 (100%)	
b) Unsupportive	3 (27.3%)	8 (72.7%)	11 (100%)	
**History of depression:**				0.542
a) Yes	14 (56%)	11 (44%)	25 (100%)	
b) No	93 (62.4%)	56 (37.6%)	149 (100%)	
**Stressful life events:**				0.003
a) Yes	32 (47.8%)	35 (52.2%)	67 (100%)	
b) No	75 (70.1%)	32 (29.9%)	107 (100%)	

*Values are given in frequency (percentage)

**P value is calculated by Pearson Chi-square test

^#^Fisher’s exact test is applied as ‘1 cells’ (25.0%) have expected count less than 5.

### 3.5 Association of PPD with maternal & child risk factors

No significant difference was found between the occurrences of PPD and maternal and child risk factors (Table 3). However, a close association was noted between mode of delivery and presence of PPD in mothers with a *P* value of 0.107 as 45% of the study population with Caesarean had PPD. Also, 40% of the mothers in post-partum phase beyond 4 weeks category had PPD, while, only 34.8% of them in early phase group had depression showing a less possibility of blue baby syndrome than PPD. There is high tendency of depression seen in mothers who have delivered more than three times (44%). Also, breastfeeding mothers had less occurrence of PPD compared to non-breast feeding mothers in their respective cohorts (36.6% Vs 45%).

**Table 3:** Association between postpartum depression and maternal and child risk factors.

Maternal and child risk factors	Normal*	Depression*	Total*	*P* value**
**Mode of delivery:**				0.107
a) Normal	66 (66.7%)	33 (33.3%)	99 (100%)	
b) Caesarean	41 (54.7%)	34 (45.3%)	75 (100%)	
**Post-partum phase:**				0.545
a) phase 1 (1–4 week postpartum)	30 (66.2%)	16 (34.8%)	46 (100%)	
b) other phases (> 4 weeks upto 24 weeks postpartum)	77 (60.2%)	51 (39.8%)	128 (100%)	
**Pregnancy:**				0.825
a) Wanted	83 (61.9%)	51 (38.1%)	134 (100%)	
b) Unwanted	24 (60%)	16 (40%)	40 (100%)	
**Delivery number:**				0.542
a) ≤ three	93 (62.4%)	56 (37.6%)	149 (100%)	
b) > three	14 (56%)	11 (44%)	25 (100%)	
**Breast feeding:**				0.336
a) No	22 (55%)	18 (45%)	40 (100%)	
b) Yes	85 (63.4%)	49 (36.6%)	134 (100%)	
**Contraception use:**				0.740
a) Yes	65 (62.5%)	39 (37.5%)	104 (100%)	
b) No	42 (60%)	28 (40%)	70 (100%)	

*Values are given in frequency (percentage)

**P value is calculated by Pearson Chi-square test

### 3.6 Predictors of PPD

Table 4 shows results of a logistic regression model. All socio-demographic, psychosocial characteristics, maternal, and child risk factors were entered in the system, then odds ratio was calculated. The predictors of PPD were unsupportive spouse (OR = 4.53, *P* = 0.049), stressful life events (OR = 2.677, *P =* 0.005), and Caesarean section as a mode of delivery (OR = 1.958, *P* = 0.049)

**Table 4:** Significant predictors of depression.

Variable	Odds ratio	95% Confidence Interval		*P* Value
		Lower	Upper	
**Stressful life events:** a) No **b)** Yes	2.677	1.344	5.333	0.005
**Spouse:** a) Supportive **b)** Non supportive	4.531	1.009	20.347	0.049
**Mode of delivery:** a) Normal b) Caesarean	1.958	1.002	3.824	0.049

## 4. Discussion

This research was a cross sectional study focused on elucidating the prevalence and predictors of postpartum depression amongst residents of Riyadh region of Saudi Arabia using established validated pretested questionnaire with Edinburgh postnatal depression scale (EPDS). The outcome of the research emphasized importance of corrective measures to curb the menace of depression and promote healthy life for mothers to facilitate proper growth and development of the new born.

The prevalence of PPD reported in this study is relatively high (38.5%) when compared to other studies of the region [30, 44]. However, our findings are in accordance with the report of World Health Organization’s (2009) that indicated 20–40% prevalence of PPD in developing countries. The possible reasons for earlier reports on lower PPD prevalence could be due to single centered sample collection [30] or limiting the interview time to 8–12 weeks post-delivery [29]. Our study was spread across several health care centers of Riyadh to have better representation of the residents of Riyadh. Also, studies involving few weeks postpartum (upto one month) may reflect only blue baby syndrome, a transient depressive phase, rather than actual depression. As this study was aimed to explore the actual prevalence of postpartum depression over extended period, the collection of data was done for 24 weeks. Additionally, among the study population, higher trend of depression was noted after the end of blue baby syndrome phase (first four weeks) indicating the increased incidence of depression in late postpartum phase. The consequences of PPD make a serious health concern as it may decrease the maternal confidence and may interfere in the growth and mental as well as physical development of infant. Therefore it is imperative to explore modifiable or controllable factors that can alleviate the risk of depression.

The strongest predictor of PPD recorded by this study was recent stressful life events. As the samples recruited in this study were in proportion to the usual flow of patients by convenient sampling, having high percentage of surveyors with stress life event might be an indication of situation of general population of Riyadh residents. The stressful life events are discrete quantifiable situation that can have a severe negative impact on psychological status which can increase the risk of depression [45]. The impact of stressful life event on the development and exacerbation of somatic disorders [46], chronic illness are well described [47]. Since the mother’s body undergoes series of changes during the process of gestation and parturition, presence of stressful life event may work as trigger for developing depression. Further, after the birth of the baby, mothers will have many new responsibilities and challenges that she ought to cope with, and being stressed by other problems will increase the load on the mother making it difficult for her to adapt with all these obstacles. Our findings were similar to the findings of another study that demonstrated the impact of stressful events and hard times on developing major depression [48].

It is well established in scientific research that induction of depression is avoidable with favorable and positive emotional support from family and community. Family support and care is vital for the wellbeing of patients undergoing physical trauma, chronic illness and some any physiological complication in life. Patients’ with depression. A study suggested to focus more on the benefits of spouse support than to the therapeutic intervention in patients under distress or prone to develop depression. [49]. Therefore life partners play an important role in preventing the onset or exacerbation of depression or any psychological manifestations that may develop during or after delivery. The stage of life during pregnancy, in general, and post-parturition, in particular is necessary to be delicately handled by the associated members of family. The supportive spouse is essential to prevent any untoward incident that may harm mother or her child. It has been found that partners who participate in daily activities, child care behavior, and helping in regular follow ups with hospitals result in reducing the onset of depression or obviating depression among their spouse. Significantly higher proportion of depression was noted in this study among mothers who reported for unsupportive life partners. This observation was in congruent with another study done elsewhere [50]. Therefore, it is imperative to disseminate importance of spouse behavior and attitude towards mothers during and after pregnancy through community teaching, health counseling and promoting family bonding.

In this study, we found mode of delivery was a key associative factor for increasing the depressions scores. High incidence of depression were noted in mothers who had C-section deliveries. A study reported earlier that that women who undergo normal vaginal delivery were at lower risk of developing depression compared to C-section cohort [51]. Postpartum women with normal deliveries might have minor-to-moderate pelvic or perennial pain. However, women who undergo C-section may have severe pain restricting their normal physical activity [52]. C-section is associated with many complications such as infections, postpartum hemorrhage, and chorionic pelvic pain that will enhance the risk of PPD [52].

## 5. Limitations

Although our research accomplished its aims, the study has also number of limitations. First, the result was based on self-reported questionnaire. Therefore, there is a chance for reporting bias, or misunderstanding of the questions. Second, Edinburgh postnatal depression scale (EPDS) includes statements about feeling and ability of doing some works by participants during the last 7 days, consequently relies on the ability to recall. Further, EPDS is only a measuring tool of depression symptoms, hence prevalence reported is just a trend and the actual occurrence of PPD may vary. Additionally, it is difficult to get assent from patients in the hospital, particularly gynecology and obstetrics department, due to cultural issues despite receiving approvals from hospital administration. This has resulted in modest sample collection. Furthermore, convenience sampling is not a true representation of the entire population. Also, longitudinal study on collection of data at different postpartum phases in the same participants would have highlighted the actual onset time of depression. Finally, there is a possibility of other predictors than those listed in the questionnaire and hence the study on predictors of PPD is not complete.

## 6. Conclusion

The prevalence of PPD among the study participants was high, especially those with recent stressful life event, unsupportive spouse and caesarean delivery. It is necessary to realize the actual existence of postpartum depression. It is not a part of essential physiological or hormonal changes they undergo after parturition. The postpartum women should be protected from this mental anomaly by advising their spouses and family members to support them throughout this experience. It is imperative to screen high-risk mothers for PPD during and after pregnancy and refer the detected cases to the community mental health centers for early management and prevention of psychosocial impairment of the family. Additionally, strategies have to be developed by health care authorities to design recommendations and actions to prevent occurrence of post-partum depression. Prevention of PPD is not only essential for wellbeing of mothers but it is important to provide good conducive atmosphere for the new born.

## Supporting information

S1 File S1_SPSS output third revision.pdf. (PDF)
