# Supplementary material for: Prevalence and predictors of postpartum depression in Riyadh, Saudi Arabia: A cross sectional study
Source: PLoS One. 2020 Feb 10;15(2):e0228666. doi: 10.1371/journal.pone.0228666 (PMC7010279; doi:10.1371/journal.pone.0228666)
Supplement: S1 File — (PDF) [file pone.0228666.s001.pdf]

```
Your temporary usage period for IBM SPSS Statistics will expire in 5828 day
s.

GET
  FILE="C:\Users\sasdag\Desktop\172\Research seminar\students' research\PPD
 project\PLOS\PLOS third revision\modified SPSS file.sav".
DATASET NAME DataSet1 WINDOW=FRONT.
FREQUENCIES VARIABLES=age_range Phase_range EMPLOYME SPOUSE PREGNANC DELIVE
RY DEPRESSI LACTATIO
    CONTRACE STRESS Depression Education_range income_range delivery_range
  /ORDER=ANALYSIS.
```

Frequencies

Notes

Output Created		16-JAN-2020 14:16:28
Comments		
Input	Data	C:\Users\sasdag\Desktop\172\Research seminar\students' research\PPD project\PLOS\PLOS third revision\modified SPSS file.sav
	Active Dataset	DataSet1
	Filter	<none>
	Weight	<none>
	Split File	<none>
	N of Rows in Working Data File	174
Missing Value Handling	Definition of Missing	User-defined missing values are treated as missing.
	Cases Used	Statistics are based on all cases with valid data.

Notes

Syntax		FREQUENCIES VARIABLES=age_range Phase_range EMPLOYME SPOUSE PREGNANC DELIVERY DEPRESSI LACTATIO<br>CONTRACE STRESS Depression Education_range income_range delivery_range<br>/ORDER=ANALYSIS.
Resources	Processor Time	00:00:00.00
	Elapsed Time	00:00:00.16

```
[DataSet1] C:\Users\sasdag\Desktop\172\Research seminar\students' research\PPD project\PLOS\PLOS third revision\modified SPSS file.sav
```

Statistics

		age_range	Phase_range	Employment status	SPOUSE	PREGNANC
N	Valid	174	174	174	174	174
	Missing	0	0	0	0	0

Statistics

		Type of delivery	History of depression	Breast feeding	Contraception use	Stressful life events
N	Valid	174	174	174	174	174
	Missing	0	0	0	0	0

Statistics

		Depression	Range of education	range of income	number of delivery range
N	Valid	174	174	174	174
	Missing	0	0	0	0

Frequency Table

age_range

		Frequency	Percent	Valid Percent	Cumulative Percent
Valid	16-25	59	33.9	33.9	33.9
	More than 25	115	66.1	66.1	100.0
	Total	174	100.0	100.0	

Phase_range

		Frequency	Percent	Valid Percent	Cumulative Percent
Valid	Phase 1	46	26.4	26.4	26.4
	Other phases	128	73.6	73.6	100.0
	Total	174	100.0	100.0	

Employment status

		Frequency	Percent	Valid Percent	Cumulative Percent
Valid	yes	38	21.8	21.8	21.8
	no	136	78.2	78.2	100.0
	Total	174	100.0	100.0	

SPOUSE

		Frequency	Percent	Valid Percent	Cumulative Percent
Valid	Supportive	163	93.7	93.7	93.7
	Unsupportive	11	6.3	6.3	100.0
	Total	174	100.0	100.0	

PREGNANC

		Frequency	Percent	Valid Percent	Cumulative Percent
Valid	Wanted	134	77.0	77.0	77.0
	Unwanted	40	23.0	23.0	100.0
	Total	174	100.0	100.0	

Type of delivery

		Frequency	Percent	Valid Percent	Cumulative Percent
Valid	Normal	99	56.9	56.9	56.9
	Caesarean	75	43.1	43.1	100.0
	Total	174	100.0	100.0	

History of depression

		Frequency	Percent	Valid Percent	Cumulative Percent
Valid	No	149	85.6	85.6	85.6
	Yes	25	14.4	14.4	100.0
	Total	174	100.0	100.0	

Breast feeding

		Frequency	Percent	Valid Percent	Cumulative Percent
Valid	No	40	23.0	23.0	23.0
	yes	134	77.0	77.0	100.0
	Total	174	100.0	100.0	

Contraception use

		Frequency	Percent	Valid Percent	Cumulative Percent
Valid	Yes	104	59.8	59.8	59.8
	No	70	40.2	40.2	100.0
	Total	174	100.0	100.0	

Stressful life events

		Frequency	Percent	Valid Percent	Cumulative Percent
Valid	No	107	61.5	61.5	61.5
	Yes	67	38.5	38.5	100.0
	Total	174	100.0	100.0	

Depression

		Frequency	Percent	Valid Percent	Cumulative Percent
Valid	Normal	107	61.5	61.5	61.5
	Depression	67	38.5	38.5	100.0
	Total	174	100.0	100.0	

Range of education

		Frequency	Percent	Valid Percent	Cumulative Percent
Valid	high education	101	58.0	58.0	58.0
	low education	73	42.0	42.0	100.0
	Total	174	100.0	100.0	

range of income

		Frequency	Percent	Valid Percent	Cumulative Percent
Valid	more than 8000	65	37.4	37.4	37.4
	less than or equal to 8000	109	62.6	62.6	100.0
	Total	174	100.0	100.0	

number of delivery range

		Frequency	Percent	Valid Percent	Cumulative Percent
Valid	upto three	149	85.6	85.6	85.6
	more than three	25	14.4	14.4	100.0
	Total	174	100.0	100.0	

```
FREQUENCIES VARIABLES=PPD_measure
  /BARCHART PERCENT
  /ORDER=ANALYSIS.
```

Frequencies

Notes

Output Created		16-JAN-2020 14:17:03
Comments		
Input	Data	C:\Users\sasdag\Desktop\172\Research seminar\students' research\PPD project\PLOS\PLOS third revision\modified SPSS file.sav
	Active Dataset	DataSet1
	Filter	<none>
	Weight	<none>
	Split File	<none>
	N of Rows in Working Data File	174
Missing Value Handling	Definition of Missing	User-defined missing values are treated as missing.
	Cases Used	Statistics are based on all cases with valid data.
Syntax		FREQUENCIES VARIABLES=PPD_measure /BARCHART PERCENT /ORDER=ANALYSIS.
Resources	Processor Time	00:00:02.42
	Elapsed Time	00:00:03.21

Statistics

Total score of PPD

N	Valid	174
	Missing	0

Total score of PPD

		Frequency	Percent	Valid Percent	Cumulative Percent
Valid	.00	1	.6	.6	.6
	1.00	1	.6	.6	1.1
	2.00	4	2.3	2.3	3.4
	3.00	4	2.3	2.3	5.7
	4.00	5	2.9	2.9	8.6
	5.00	4	2.3	2.3	10.9
	6.00	9	5.2	5.2	16.1
	7.00	13	7.5	7.5	23.6
	8.00	7	4.0	4.0	27.6
	9.00	13	7.5	7.5	35.1
	10.00	12	6.9	6.9	42.0
	11.00	18	10.3	10.3	52.3
	12.00	16	9.2	9.2	61.5
	13.00	6	3.4	3.4	64.9
	14.00	9	5.2	5.2	70.1
	15.00	8	4.6	4.6	74.7
	16.00	11	6.3	6.3	81.0
	17.00	3	1.7	1.7	82.8
	18.00	7	4.0	4.0	86.8
	19.00	3	1.7	1.7	88.5
	20.00	5	2.9	2.9	91.4
	21.00	4	2.3	2.3	93.7
	22.00	4	2.3	2.3	96.0
	23.00	3	1.7	1.7	97.7
	24.00	2	1.1	1.1	98.9
	26.00	1	.6	.6	99.4
	28.00	1	.6	.6	100.0
	Total	174	100.0	100.0	

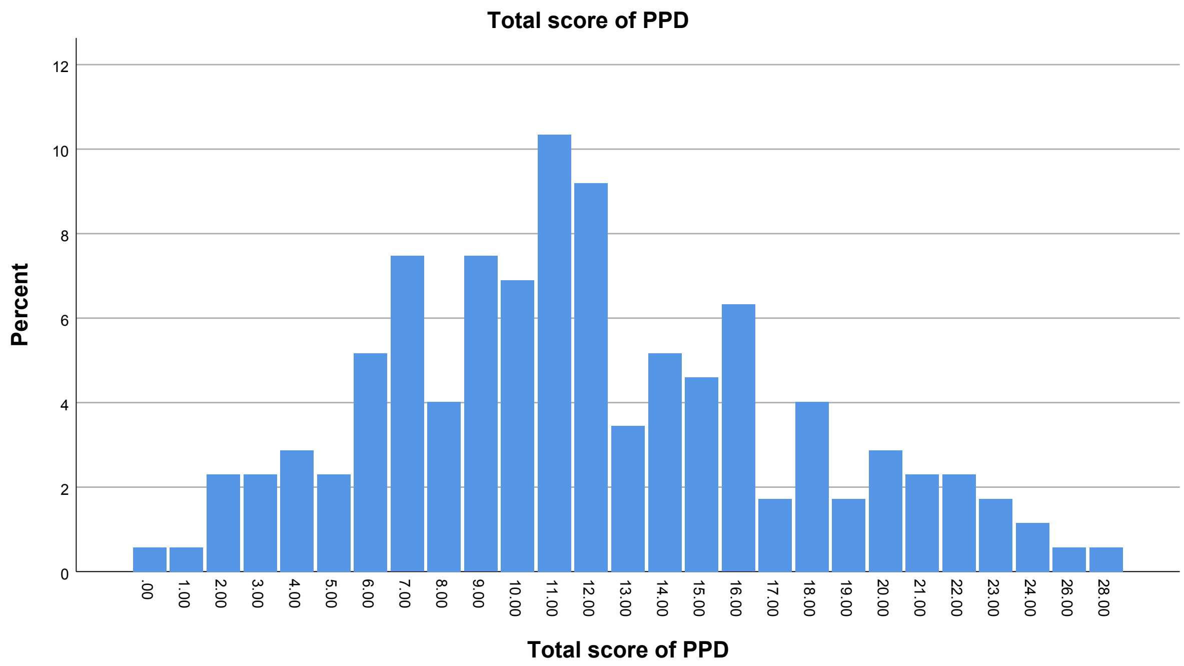


```
CROSSTABS
  /TABLES=Depression BY age_range Phase_range EMPLOYME SPOUSE PREGNANC DELI
VERY DEPRESSI LACTATIO
     CONTRACE STRESS Education_range income_range delivery_range
  /FORMAT=AVALUE TABLES
  /STATISTICS=CHISQ
  /CELLS=COUNT ROW COLUMN TOTAL
  /COUNT ROUND CELL.
```

Crosstabs

Notes

Output Created		16-JAN-2020 14:18:13
Comments		
Input	Data	C: \Users\sasdag\Desktop\172\Research seminar\students' research\PPD project\PLOS\PLOS third revision\modified SPSS file.sav
	Active Dataset	DataSet1
	Filter	<none>
	Weight	<none>
	Split File	<none>
	N of Rows in Working Data File	174
Missing Value Handling	Definition of Missing	User-defined missing values are treated as missing.
	Cases Used	Statistics for each table are based on all the cases with valid data in the specified range(s) for all variables in each table.
Syntax		CROSSTABS /TABLES=Depression BY age_range Phase_range EMPLOYME SPOUSE PREGNANC DELIVERY DEPRESSI LACTATIO CONTRACE STRESS Education_range income_range delivery_range /FORMAT=AVALUE TABLES /STATISTICS=CHISQ /CELLS=COUNT ROW COLUMN TOTAL /COUNT ROUND CELL.
Resources	Processor Time	00:00:00.02
	Elapsed Time	00:00:00.24
	Dimensions Requested	2
	Cells Available	524245

Case Processing Summary

	Cases					
	Valid		Missing		Total	
	N	Percent	N	Percent	N	Percent
Depression * age_range	174	100.0%	0	0.0%	174	100.0%
Depression * Phase_range	174	100.0%	0	0.0%	174	100.0%
Depression * Employment status	174	100.0%	0	0.0%	174	100.0%
Depression * SPOUSE	174	100.0%	0	0.0%	174	100.0%
Depression * PREGNANC	174	100.0%	0	0.0%	174	100.0%
Depression * Type of delivery	174	100.0%	0	0.0%	174	100.0%
Depression * History of depression	174	100.0%	0	0.0%	174	100.0%
Depression * Breast feeding	174	100.0%	0	0.0%	174	100.0%
Depression * Contraception use	174	100.0%	0	0.0%	174	100.0%
Depression * Stressful life events	174	100.0%	0	0.0%	174	100.0%
Depression * Range of education	174	100.0%	0	0.0%	174	100.0%
Depression * range of income	174	100.0%	0	0.0%	174	100.0%
Depression * number of delivery range	174	100.0%	0	0.0%	174	100.0%

Depression * age_range

Crosstab

			age_range		
			16-25	More than 25	Total
Depression	Normal	Count	38	69	107
		% within Depression	35.5%	64.5%	100.0%
		% within age_range	64.4%	60.0%	61.5%
		% of Total	21.8%	39.7%	61.5%
	Depression	Count	21	46	67
		% within Depression	31.3%	68.7%	100.0%
		% within age_range	35.6%	40.0%	38.5%
		% of Total	12.1%	26.4%	38.5%
Total		Count	59	115	174
		% within Depression	33.9%	66.1%	100.0%
		% within age_range	100.0%	100.0%	100.0%
		% of Total	33.9%	66.1%	100.0%

Chi-Square Tests

	Value	df	Asymptotic Significance (2-sided)	Exact Sig. (2-sided)	Exact Sig. (1-sided)
Pearson Chi-Square	.320[a]	1	.572		
Continuity Correction[b]	.161	1	.688		
Likelihood Ratio	.321	1	.571		
Fisher's Exact Test				.624	.346
Linear-by-Linear Association	.318	1	.573		
N of Valid Cases	174				

a. 0 cells (0.0%) have expected count less than 5. The minimum expected count is 22.72.

b. Computed only for a 2x2 table

Depression * Phase_range

Crosstab

			Phase_range		Total
			Phase 1	Other phases	
Depression	Normal	Count	30	77	107
		% within Depression	28.0%	72.0%	100.0%
		% within Phase_range	65.2%	60.2%	61.5%
		% of Total	17.2%	44.3%	61.5%
	Depression	Count	16	51	67
		% within Depression	23.9%	76.1%	100.0%
		% within Phase_range	34.8%	39.8%	38.5%
		% of Total	9.2%	29.3%	38.5%
Total		Count	46	128	174
		% within Depression	26.4%	73.6%	100.0%
		% within Phase_range	100.0%	100.0%	100.0%
		% of Total	26.4%	73.6%	100.0%

Chi-Square Tests

	Value	df	Asymptotic Significance (2-sided)	Exact Sig. (2-sided)	Exact Sig. (1-sided)
Pearson Chi-Square	.366[a]	1	.545		
Continuity Correction[b]	.184	1	.668		
Likelihood Ratio	.369	1	.543		
Fisher's Exact Test				.599	.336
Linear-by-Linear Association	.364	1	.546		
N of Valid Cases	174				

a. 0 cells (0.0%) have expected count less than 5. The minimum expected count is 17.71.

b. Computed only for a 2x2 table

Depression * Employment status

Crosstab

			Employment status		
			yes	no	Total
Depression	Normal	Count	23	84	107
		% within Depression	21.5%	78.5%	100.0%
		% within Employment status	60.5%	61.8%	61.5%
		% of Total	13.2%	48.3%	61.5%
	Depression	Count	15	52	67
		% within Depression	22.4%	77.6%	100.0%
		% within Employment status	39.5%	38.2%	38.5%
		% of Total	8.6%	29.9%	38.5%
Total		Count	38	136	174
		% within Depression	21.8%	78.2%	100.0%
		% within Employment status	100.0%	100.0%	100.0%
		% of Total	21.8%	78.2%	100.0%

Chi-Square Tests

	Value	df	Asymptotic Significance (2-sided)	Exact Sig. (2-sided)	Exact Sig. (1-sided)
Pearson Chi-Square	.019[a]	1	.890		
Continuity Correction[b]	.000	1	1.000		
Likelihood Ratio	.019	1	.890		
Fisher's Exact Test				1.000	.517
Linear-by-Linear Association	.019	1	.890		
N of Valid Cases	174				

a. 0 cells (0.0%) have expected count less than 5. The minimum expected count is 14.63.

b. Computed only for a 2x2 table

Depression * SPOUSE

Crosstab

			SPOUSE		Total
			Supportive	Unsupportive	
Depression	Normal	Count	104	3	107
		% within Depression	97.2%	2.8%	100.0%
		% within SPOUSE	63.8%	27.3%	61.5%
		% of Total	59.8%	1.7%	61.5%
	Depression	Count	59	8	67
		% within Depression	88.1%	11.9%	100.0%
		% within SPOUSE	36.2%	72.7%	38.5%
		% of Total	33.9%	4.6%	38.5%
Total		Count	163	11	174
		% within Depression	93.7%	6.3%	100.0%
		% within SPOUSE	100.0%	100.0%	100.0%
		% of Total	93.7%	6.3%	100.0%

Chi-Square Tests

	Value	df	Asymptotic Significance (2-sided)	Exact Sig. (2-sided)	Exact Sig. (1-sided)
Pearson Chi-Square	5.808[a]	1	.016		
Continuity Correction[b]	4.367	1	.037		
Likelihood Ratio	5.666	1	.017		
Fisher's Exact Test				.023	.019
Linear-by-Linear Association	5.774	1	.016		
N of Valid Cases	174				

a. 1 cells (25.0%) have expected count less than 5. The minimum expected count is 4.24.

b. Computed only for a 2x2 table

Depression * PREGNANC

Crosstab

			PREGNANC		
			Wanted	Unwanted	Total
Depression	Normal	Count	83	24	107
		% within Depression	77.6%	22.4%	100.0%
		% within PREGNANC	61.9%	60.0%	61.5%
		% of Total	47.7%	13.8%	61.5%
	Depression	Count	51	16	67
		% within Depression	76.1%	23.9%	100.0%
		% within PREGNANC	38.1%	40.0%	38.5%
		% of Total	29.3%	9.2%	38.5%
Total		Count	134	40	174
		% within Depression	77.0%	23.0%	100.0%
		% within PREGNANC	100.0%	100.0%	100.0%
		% of Total	77.0%	23.0%	100.0%

Chi-Square Tests

	Value	df	Asymptotic Significance (2-sided)	Exact Sig. (2-sided)	Exact Sig. (1-sided)
Pearson Chi-Square	.049[a]	1	.825		
Continuity Correction[b]	.001	1	.971		
Likelihood Ratio	.049	1	.825		
Fisher's Exact Test				.854	.482
Linear-by-Linear Association	.049	1	.825		
N of Valid Cases	174				

a. 0 cells (0.0%) have expected count less than 5. The minimum expected count is 15.40.

b. Computed only for a 2x2 table

Depression * Type of delivery

Crosstab

			Type of delivery		Total
			Normal	Caesarean	
Depression	Normal	Count	66	41	107
		% within Depression	61.7%	38.3%	100.0%
		% within Type of delivery	66.7%	54.7%	61.5%
		% of Total	37.9%	23.6%	61.5%
	Depression	Count	33	34	67
		% within Depression	49.3%	50.7%	100.0%
		% within Type of delivery	33.3%	45.3%	38.5%
		% of Total	19.0%	19.5%	38.5%
Total		Count	99	75	174
		% within Depression	56.9%	43.1%	100.0%
		% within Type of delivery	100.0%	100.0%	100.0%
		% of Total	56.9%	43.1%	100.0%

Chi-Square Tests

	Value	df	Asymptotic Significance (2-sided)	Exact Sig. (2-sided)	Exact Sig. (1-sided)
Pearson Chi-Square	2.595[a]	1	.107		
Continuity Correction[b]	2.113	1	.146		
Likelihood Ratio	2.589	1	.108		
Fisher's Exact Test				.118	.073
Linear-by-Linear Association	2.580	1	.108		
N of Valid Cases	174				

a. 0 cells (0.0%) have expected count less than 5. The minimum expected count is 28.88.

b. Computed only for a 2x2 table

Depression * History of depression

Crosstab

			History of depression		Total
			No	Yes	
Depression	Normal	Count	93	14	107
		% within Depression	86.9%	13.1%	100.0%
		% within History of depression	62.4%	56.0%	61.5%
		% of Total	53.4%	8.0%	61.5%
	Depression	Count	56	11	67
		% within Depression	83.6%	16.4%	100.0%
		% within History of depression	37.6%	44.0%	38.5%
		% of Total	32.2%	6.3%	38.5%
Total		Count	149	25	174
		% within Depression	85.6%	14.4%	100.0%
		% within History of depression	100.0%	100.0%	100.0%
		% of Total	85.6%	14.4%	100.0%

Chi-Square Tests

	Value	df	Asymptotic Significance (2-sided)	Exact Sig. (2-sided)	Exact Sig. (1-sided)
Pearson Chi-Square	.372[a]	1	.542		
Continuity Correction[b]	.151	1	.698		
Likelihood Ratio	.367	1	.544		
Fisher's Exact Test				.658	.345
Linear-by-Linear Association	.370	1	.543		
N of Valid Cases	174				

a. 0 cells (0.0%) have expected count less than 5. The minimum expected count is 9.63.

b. Computed only for a 2x2 table

Depression * Breast feeding

Crosstab

			Breast feeding		
			No	yes	Total
Depression	Normal	Count	22	85	107
		% within Depression	20.6%	79.4%	100.0%
		% within Breast feeding	55.0%	63.4%	61.5%
		% of Total	12.6%	48.9%	61.5%
	Depression	Count	18	49	67
		% within Depression	26.9%	73.1%	100.0%
		% within Breast feeding	45.0%	36.6%	38.5%
		% of Total	10.3%	28.2%	38.5%
Total		Count	40	134	174
		% within Depression	23.0%	77.0%	100.0%
		% within Breast feeding	100.0%	100.0%	100.0%
		% of Total	23.0%	77.0%	100.0%

Chi-Square Tests

	Value	df	Asymptotic Significance (2-sided)	Exact Sig. (2-sided)	Exact Sig. (1-sided)
Pearson Chi-Square	.925[a]	1	.336		
Continuity Correction[b]	.603	1	.437		
Likelihood Ratio	.914	1	.339		
Fisher's Exact Test				.359	.218
Linear-by-Linear Association	.920	1	.338		
N of Valid Cases	174				

a. 0 cells (0.0%) have expected count less than 5. The minimum expected count is 15.40.

b. Computed only for a 2x2 table

Depression * Contraception use

Crosstab

			Contraception use		Total
			Yes	No	
Depression	Normal	Count	65	42	107
		% within Depression	60.7%	39.3%	100.0%
		% within Contraception use	62.5%	60.0%	61.5%
		% of Total	37.4%	24.1%	61.5%
	Depression	Count	39	28	67
		% within Depression	58.2%	41.8%	100.0%
		% within Contraception use	37.5%	40.0%	38.5%
		% of Total	22.4%	16.1%	38.5%
Total		Count	104	70	174
		% within Depression	59.8%	40.2%	100.0%
		% within Contraception use	100.0%	100.0%	100.0%
		% of Total	59.8%	40.2%	100.0%

Chi-Square Tests

	Value	df	Asymptotic Significance (2-sided)	Exact Sig. (2-sided)	Exact Sig. (1-sided)
Pearson Chi-Square	.110[a]	1	.740		
Continuity Correction[b]	.030	1	.862		
Likelihood Ratio	.110	1	.740		
Fisher's Exact Test				.753	.430
Linear-by-Linear Association	.110	1	.740		
N of Valid Cases	174				

a. 0 cells (0.0%) have expected count less than 5. The minimum expected count is 26.95.

b. Computed only for a 2x2 table

Depression * Stressful life events

Crosstab

			Stressful life events		Total
			No	Yes	
Depression	Normal	Count	75	32	107
		% within Depression	70.1%	29.9%	100.0%
		% within Stressful life events	70.1%	47.8%	61.5%
		% of Total	43.1%	18.4%	61.5%
	Depression	Count	32	35	67
		% within Depression	47.8%	52.2%	100.0%
		% within Stressful life events	29.9%	52.2%	38.5%
		% of Total	18.4%	20.1%	38.5%
Total		Count	107	67	174
		% within Depression	61.5%	38.5%	100.0%
		% within Stressful life events	100.0%	100.0%	100.0%
		% of Total	61.5%	38.5%	100.0%

Chi-Square Tests

	Value	df	Asymptotic Significance (2-sided)	Exact Sig. (2-sided)	Exact Sig. (1-sided)
Pearson Chi-Square	8.678[a]	1	.003		
Continuity Correction[b]	7.760	1	.005		
Likelihood Ratio	8.635	1	.003		
Fisher's Exact Test				.004	.003
Linear-by-Linear Association	8.628	1	.003		
N of Valid Cases	174				

a. 0 cells (0.0%) have expected count less than 5. The minimum expected count is 25.80.

b. Computed only for a 2x2 table

Depression * Range of education

Crosstab

			Range of education		
			high education	low education	Total
Depression	Normal	Count	61	46	107
		% within Depression	57.0%	43.0%	100.0%
		% within Range of education	60.4%	63.0%	61.5%
		% of Total	35.1%	26.4%	61.5%
	Depression	Count	40	27	67
		% within Depression	59.7%	40.3%	100.0%
		% within Range of education	39.6%	37.0%	38.5%
		% of Total	23.0%	15.5%	38.5%
Total		Count	101	73	174
		% within Depression	58.0%	42.0%	100.0%
		% within Range of education	100.0%	100.0%	100.0%
		% of Total	58.0%	42.0%	100.0%

Chi-Square Tests

	Value	df	Asymptotic Significance (2-sided)	Exact Sig. (2-sided)	Exact Sig. (1-sided)
Pearson Chi-Square	.123[a]	1	.726		
Continuity Correction[b]	.037	1	.847		
Likelihood Ratio	.123	1	.726		
Fisher's Exact Test				.754	.425
Linear-by-Linear Association	.122	1	.727		
N of Valid Cases	174				

a. 0 cells (0.0%) have expected count less than 5. The minimum expected count is 28.11.

b. Computed only for a 2x2 table

Depression * range of income

Crosstab

			range of income		
			more than 8000	less than or equal to 8000	Total
Depression	Normal	Count	42	65	107
		% within Depression	39.3%	60.7%	100.0%
		% within range of income	64.6%	59.6%	61.5%
		% of Total	24.1%	37.4%	61.5%
	Depression	Count	23	44	67
		% within Depression	34.3%	65.7%	100.0%
		% within range of income	35.4%	40.4%	38.5%
		% of Total	13.2%	25.3%	38.5%
Total		Count	65	109	174
		% within Depression	37.4%	62.6%	100.0%
		% within range of income	100.0%	100.0%	100.0%
		% of Total	37.4%	62.6%	100.0%

Chi-Square Tests

	Value	df	Asymptotic Significance (2-sided)	Exact Sig. (2-sided)	Exact Sig. (1-sided)
Pearson Chi-Square	.427[a]	1	.514		
Continuity Correction[b]	.242	1	.622		
Likelihood Ratio	.429	1	.513		
Fisher's Exact Test				.525	.312
Linear-by-Linear Association	.424	1	.515		
N of Valid Cases	174				

a. 0 cells (0.0%) have expected count less than 5. The minimum expected count is 25.03.

b. Computed only for a 2x2 table

Depression * number of delivery range

Crosstab

			number of delivery range		
			upto three	more than three	Total
Depression	Normal	Count	93	14	107
		% within Depression	86.9%	13.1%	100.0%
		% within number of delivery range	62.4%	56.0%	61.5%
		% of Total	53.4%	8.0%	61.5%
	Depression	Count	56	11	67
		% within Depression	83.6%	16.4%	100.0%
		% within number of delivery range	37.6%	44.0%	38.5%
		% of Total	32.2%	6.3%	38.5%
Total		Count	149	25	174
		% within Depression	85.6%	14.4%	100.0%
		% within number of delivery range	100.0%	100.0%	100.0%
		% of Total	85.6%	14.4%	100.0%

Chi-Square Tests

	Value	df	Asymptotic Significance (2-sided)	Exact Sig. (2-sided)	Exact Sig. (1-sided)
Pearson Chi-Square	.372[a]	1	.542		
Continuity Correction[b]	.151	1	.698		
Likelihood Ratio	.367	1	.544		
Fisher's Exact Test				.658	.345
Linear-by-Linear Association	.370	1	.543		
N of Valid Cases	174				

a. 0 cells (0.0%) have expected count less than 5. The minimum expected count is 9.63.

b. Computed only for a 2x2 table

```
LOGISTIC REGRESSION VARIABLES Depression
  /METHOD=ENTER age_range Phase_range EMPLOYME SPOUSE PREGNANC DELIVERY DEP
RESSI LACTATIO CONTRACE
    STRESS Education_range income_range delivery_range
  /CRITERIA=PIN(0.05) POUT(0.10) ITERATE(20) CUT(0.5).
```

Logistic Regression

Notes

Output Created		16-JAN-2020 14:28:27
Comments		
Input	Data	C: \Users\sasdag\Desktop\172\Research seminar\students' research\PPD project\PLOS\PLOS third revision\modified SPSS file.sav
	Active Dataset	DataSet1
	Filter	<none>
	Weight	<none>
	Split File	<none>
	N of Rows in Working Data File	174
Missing Value Handling	Definition of Missing	User-defined missing values are treated as missing
Syntax		LOGISTIC REGRESSION VARIABLES Depression /METHOD=ENTER age_range Phase_range EMPLOYME SPOUSE PREGNANC DELIVERY DEPRESSI LACTATIO CONTRACE STRESS Education_range income_range delivery_range /CRITERIA=PIN(0.05) POUT(0.10) ITERATE(20) CUT(0.5).
Resources	Processor Time	00:00:00.03
	Elapsed Time	00:00:00.06

Case Processing Summary

Unweighted Cases[a]		N	Percent
Selected Cases	Included in Analysis	174	100.0
	Missing Cases	0	.0
	Total	174	100.0
Unselected Cases		0	.0
Total		174	100.0

a. If weight is in effect, see classification table for the total number of cases.

Dependent Variable Encoding

Original Value	Internal Value
Normal	0
Depression	1

Block 0: Beginning Block

Classification Table[a,b]

Observed			Predicted		
			Depression		Percentage Correct
			Normal	Depression	
Step 0	Depression	Normal	107	0	100.0
		Depression	67	0	.0
	Overall Percentage				61.5

a. Constant is included in the model.

b. The cut value is .500

Variables in the Equation

		B	S.E.	Wald	df	Sig.	Exp(B)
Step 0	Constant	-.468-	.156	9.029	1	.003	.626

Variables not in the Equation

			Score	df	Sig.
Step 0	Variables	age_range	.320	1	.572
		Phase_range	.366	1	.545
		Employment status	.019	1	.890
		SPOUSE	5.808	1	.016
		PREGNANC	.049	1	.825
		Type of delivery	2.595	1	.107
		History of depression	.372	1	.542
		Breast feeding	.925	1	.336
		Contraception use	.110	1	.740
		Stressful life events	8.678	1	.003
		Range of education	.123	1	.726
		range of income	.427	1	.514
		number of delivery range	.372	1	.542
	Overall Statistics		18.902	13	.126

Block 1: Method = Enter

Omnibus Tests of Model Coefficients

		Chi-square	df	Sig.
Step 1	Step	19.574	13	.106
	Block	19.574	13	.106
	Model	19.574	13	.106

Model Summary

Step	-2 Log likelihood	Cox & Snell R Square	Nagelkerke R Square
1	212.363[a]	.106	.145

a. Estimation terminated at iteration number 4 because parameter estimates changed by less than .001.

Classification Table[a]

Observed			Predicted: Depression: Normal	Predicted: Depression: Depression	Predicted: Percentage Correct
Step 1	Depression	Normal	95	12	88.8
		Depression	41	26	38.8
	Overall Percentage				69.5

a. The cut value is .500

Variables in the Equation

		B	S.E.	Wald	df	Sig.
Step 1[a]	age_range	-.006-	.374	.000	1	.988
	Phase_range	.310	.409	.575	1	.448
	Employment status	.220	.451	.239	1	.625
	SPOUSE	1.511	.766	3.886	1	.049
	PREGNANC	-.303-	.425	.507	1	.477
	Type of delivery	.672	.342	3.869	1	.049
	History of depression	.259	.491	.278	1	.598
	Breast feeding	-.306-	.396	.599	1	.439
	Contraception use	.230	.348	.435	1	.510
	Stressful life events	.985	.352	7.841	1	.005
	Range of education	-.204-	.352	.335	1	.562
	range of income	.055	.356	.024	1	.877
	number of delivery range	.417	.537	.604	1	.437
	Constant	-2.361-	1.373	2.955	1	.086

Variables in the Equation

		Exp(B)
Step 1[a]	age_range	.994
	Phase_range	1.364
	Employment status	1.246
	SPOUSE	4.531
	PREGNANC	.739
	Type of delivery	1.958
	History of depression	1.295
	Breast feeding	.736
	Contraception use	1.258
	Stressful life events	2.677
	Range of education	.816
	range of income	1.057
	number of delivery range	1.518
	Constant	.094

a. Variable(s) entered on step 1: age_range, Phase_range, Employment status, SPOUSE, PREGNANC, Type of delivery, History of depression, Breast feeding, Contraception use, Stressful life events, Range of education, range of income, number of delivery range.

```
 LOGISTIC REGRESSION VARIABLES Depression
  /METHOD=ENTER age_range Phase_range EMPLOYME SPOUSE PREGNANC DELIVERY DEP
RESSI LACTATIO CONTRACE
```

```
  STRESS Education_range income_range delivery_range
/PRINT=CI(95)
/CRITERIA=PIN(0.05) POUT(0.10) ITERATE(20) CUT(0.5).
```

Logistic Regression

Notes

Output Created		16-JAN-2020 14:31:03
Comments		
Input	Data	C: \Users\sasdag\Desktop\172\Research seminar\students' research\PPD project\PLOS\PLOS third revision\modified SPSS file.sav
	Active Dataset	DataSet1
	Filter	<none>
	Weight	<none>
	Split File	<none>
	N of Rows in Working Data File	174
Missing Value Handling	Definition of Missing	User-defined missing values are treated as missing
Syntax		LOGISTIC REGRESSION VARIABLES Depression /METHOD=ENTER age_range Phase_range EMPLOYME SPOUSE PREGNANC DELIVERY DEPRESSI LACTATIO CONTRACE STRESS Education_range income_range delivery_range /PRINT=CI(95) /CRITERIA=PIN(0.05) POUT(0.10) ITERATE(20) CUT(0.5).
Resources	Processor Time	00:00:00.00
	Elapsed Time	00:00:00.02

Case Processing Summary

Unweighted Cases[a]		N	Percent
Selected Cases	Included in Analysis	174	100.0
	Missing Cases	0	.0
	Total	174	100.0
Unselected Cases		0	.0
Total		174	100.0

a. If weight is in effect, see classification table for the total number of cases.

Dependent Variable Encoding

Original Value	Internal Value
Normal	0
Depression	1

Block 0: Beginning Block

Classification Table[a,b]

	Observed		Predicted		
			Depression		Percentage Correct
			Normal	Depression	
Step 0	Depression	Normal	107	0	100.0
		Depression	67	0	.0
	Overall Percentage				61.5

a. Constant is included in the model.

b. The cut value is .500

Variables in the Equation

		B	S.E.	Wald	df	Sig.	Exp(B)
Step 0	Constant	-.468-	.156	9.029	1	.003	.626

Variables not in the Equation

			Score	df	Sig.
Step 0	Variables	age_range	.320	1	.572
		Phase_range	.366	1	.545
		Employment status	.019	1	.890
		SPOUSE	5.808	1	.016
		PREGNANC	.049	1	.825
		Type of delivery	2.595	1	.107
		History of depression	.372	1	.542
		Breast feeding	.925	1	.336
		Contraception use	.110	1	.740
		Stressful life events	8.678	1	.003
		Range of education	.123	1	.726
		range of income	.427	1	.514
		number of delivery range	.372	1	.542
	Overall Statistics		18.902	13	.126

Block 1: Method = Enter

Omnibus Tests of Model Coefficients

		Chi-square	df	Sig.
Step 1	Step	19.574	13	.106
	Block	19.574	13	.106
	Model	19.574	13	.106

Model Summary

Step	-2 Log likelihood	Cox & Snell R Square	Nagelkerke R Square
1	212.363[a]	.106	.145

a. Estimation terminated at iteration number 4 because parameter estimates changed by less than .001.

Classification Table[a]

Observed			Predicted: Depression Normal	Predicted: Depression Depression	Percentage Correct
Step 1	Depression	Normal	95	12	88.8
		Depression	41	26	38.8
	Overall Percentage				69.5

a. The cut value is .500

Variables in the Equation

		B	S.E.	Wald	df	Sig.
Step 1[a]	age_range	-.006-	.374	.000	1	.988
	Phase_range	.310	.409	.575	1	.448
	Employment status	.220	.451	.239	1	.625
	SPOUSE	1.511	.766	3.886	1	.049
	PREGNANC	-.303-	.425	.507	1	.477
	Type of delivery	.672	.342	3.869	1	.049
	History of depression	.259	.491	.278	1	.598
	Breast feeding	-.306-	.396	.599	1	.439
	Contraception use	.230	.348	.435	1	.510
	Stressful life events	.985	.352	7.841	1	.005
	Range of education	-.204-	.352	.335	1	.562
	range of income	.055	.356	.024	1	.877
	number of delivery range	.417	.537	.604	1	.437
	Constant	-2.361-	1.373	2.955	1	.086

Variables in the Equation

		Exp(B)	95% C.I.for EXP(B)	
			Lower	Upper
Step 1[a]	age_range	.994	.478	2.068
	Phase_range	1.364	.612	3.039
	Employment status	1.246	.515	3.016
	SPOUSE	4.531	1.009	20.347
	PREGNANC	.739	.321	1.700
	Type of delivery	1.958	1.002	3.824
	History of depression	1.295	.495	3.388
	Breast feeding	.736	.339	1.599
	Contraception use	1.258	.636	2.490
	Stressful life events	2.677	1.344	5.333
	Range of education	.816	.410	1.625
	range of income	1.057	.526	2.124
	number of delivery range	1.518	.530	4.349
	Constant	.094		

a. Variable(s) entered on step 1: age_range, Phase_range, Employment status, SPOUSE, PREGNANC, Type of delivery, History of depression, Breast feeding, Contraception use, Stressful life events, Range of education, range of income, number of delivery range.
